# Ultrasound as a Rapid and Low-Cost Extraction Procedure to Obtain Anthocyanin-Based Colorants from *Prunus spinosa* L. Fruit Epicarp: Comparative Study with Conventional Heat-Based Extraction

**DOI:** 10.3390/molecules24030573

**Published:** 2019-02-05

**Authors:** Maria G. Leichtweis, Carla Pereira, M.A. Prieto, Maria Filomena Barreiro, Ilton José Baraldi, Lillian Barros, Isabel C.F.R. Ferreira

**Affiliations:** 1 Centro de Investigação de Montanha (CIMO), Instituto Politécnico de Bragança, Campus de Santa Apolónia, 5300-253 Bragança, Portugal; mg.leichtweis@hotmail.com (M.G.L.); carlap@ipb.pt (C.P.); michaelumangelum@gmail.com (M.A.P.); barreiro@ipb.pt (M.F.B.); 2 Nutrition and Food Science Group, Department of Analytical and Food Chemistry, CITACA, CACTI, University of Vigo-Vigo Campus, 36310 Vigo, Spain; 3 Laboratory of Separation and Reaction Engineering—Laboratory of Catalysis and Materials (LSRE-LCM), Polytechnic Institute of Bragança, Campus Santa Apolónia, 5300-253 Bragança, Portugal; 4 Departamento Acadêmico de Alimentos (DAALM), Universidade Tecnológica Federal do Paraná, Campus Medianeira, 85884-000 Paraná, Brasil; ijbaraldi@gmail.com

**Keywords:** *Prunus spinosa* L. fruit epicarp, wild fruit valorization, cyanidin 3-rutinoside, peonidin 3-rutinoside, heat and ultrasound assisted extraction, response surface methodology

## Abstract

An ultrasound rapid and low-cost procedure for anthocyanin-based colorants from *Prunus spinosa* L. fruit epicarp was developed, and the advantages were compared with conventional heat-based extraction. To obtain the conditions that maximize anthocyanins’ extraction, a response surface methodology was applied using the variables of time, temperature, and ethanol content, in the case of heat extraction, whereas for ultrasound assisted extraction, temperature was replaced by ultrasound power. Two anthocyanin compounds were identified by HPLC-DAD-ESI/MS—namely, cyanidin 3-rutinoside and peonidin 3-rutinoside. The responses used were the extraction yield and the content of the identified anthocyanins. Ultrasound extraction was the most effective method at 5.00 ± 0.15 min, 400.00 ± 32.00 W, and 47.98% ± 2.88% of ethanol obtaining 68.60% ± 2.06% of extracted residue, with an anthocyanin content of 18.17 mg/g (extract-basis) and 11.76 mg/g (epicarp-basis). Overall, a viable green process was achieved that could be used to support pilot-scale studies for industrial production of anthocyanin-based colorants from *P. spinosa* fruit epicarp.

## 1. Introduction

*Prunus spinosa* L. (blackthorn) is a spontaneous wild shrub found in Portugal, Spain, and other European countries. Its fruits are commonly used for liqueur and jam preparations, as well as for medicinal purposes [1]. Nevertheless, no reports were found regarding the industrial, or large scale, use of these fruits, probably because of their bitter and astringent taste.

The valorization of agricultural products has gained much attention in the late years as a mean for a sustainable management, which can concomitantly increase the profit of local economies. In this regard, *P. spinosa* constitutes an underexploited source and can serve as a raw material for the recovery/production of compounds for food applications [2]. As with other *Prunus* species, anthocyanin compounds can be found in blackthorn fruits at high levels, being responsible for their typical coloration [3,4]. In fact, a complex profile of anthocyanins was previously identified in *P. spinosa* fruits, among which cyanidin 3-rutinoside and peonidin 3-rutinoside were found to be predominant [5,6].

Anthocyanins are natural pigments belonging to the phenolic compounds group and, within that, to the flavonoids class, presenting a range of colors between red, blue, and violet that are characteristic of various fruits and vegetables [7]. Beyond their various physiological benefits, which include effects against cardiovascular diseases, atherosclerosis, and cancer, recently, an increasing interest in these compounds began to arise because of their colorant properties [8,9].

The industrial production of natural-based colorants has been established for years and consists mainly of obtaining colorant-rich extracts through conventional heat assisted extraction (HAE, or maceration) using water as a solvent followed by several isolation/drying steps. This type of conventional process, although used at large-scale, is known for requiring high-energy consumption and long extraction times [10,11,12]. Alternative extraction processes, able to replace traditional ones, have been established to shorten the needed time, decrease energy requirements, and reduce solvent consumption. Among the non-conventional procedures applied to anthocyanins’ extraction, ultrasound, microwave, and supercritical fluid assisted extraction techniques have attracted, in the recent years, the attention of industrials and researchers [10,13]. Regarding ultrasound assisted extraction (UAE), it is considered an inexpensive, simple, and efficient alternative to conventional techniques [14]. The extraction capability of UAE is attributed to mechanical and cavitation phenomena, which lead to cells’ disruption, particle size reduction, and enhanced mass transfer across the cell membrane [11,13].

To obtain anthocyanin-rich extracts, it is crucial to consider the factors affecting the stability of these compounds, including structure and concentration, pH, temperature, light exposure, oxygen levels, and used extraction solvents [15]. Thus, the choice of the extraction method, along with the optimization of relevant extraction variables, are essential to guarantee a maximum recovery efficiency [16]. Additionally, the efficiency is also strongly affected by the variability observed among different matrices [17]. Through response surface methodology (RSM), it is possible to optimize the relevant variables simultaneously, obtaining polynomial models capable of describing, within the tested experimental interval, the ideal conditions that maximize the used response criteria [13].

In the present study, the goal was to explore blackthorn anthocyanin composition and promote a higher commercial value of these wild fruits through the development of an anthocyanin-based coloring extract. For that purpose, the fruit epicarp was used because it has a much more intense color than the pulp, and thus a higher concentration of anthocyanins and less interfering compounds in the extraction process (e.g., sugars). To the best of our knowledge, and according to a thorough literature survey, no reference or report on the optimization of anthocyanin compounds extraction from fruit epicarps of *P. spinose* was found. Therefore, the present study aimed to optimize the extraction of these compounds from *P. spinosa* fruit epicarps through HAE and UAE techniques, evaluating the following variables: i) type of solvent (water and green organic solvents); ii) extraction time; iii) solid-to-liquid ratio; and iv) temperature (for HAE) or pressure (for UAE). The most efficient parameters were obtained by response surface methodology (RSM). The identification and quantification of the anthocyanin compounds present in the extracts was assessed by HPLC-DAD-ESI/MS.

## 2. Results

### 2.1. Development of RSM Models to Optimize Responses and Conditions

The RSM is a valuable instrument to assess the impact of the main extraction factors and their interactions on one or more responses. The technique uses fixed experimental designs with the major goals of minimizing the experimental labor and finding optimal solutions. In this regard, the work presented here uses the *circumscribed central composite design (CCCD)* design plan, which is a popular design among researchers when trying to optimize food processing methods [18], such as the extraction of anthocyanin compounds.

In a previous study [5], authors identified, using HPLC-DAD-ESI/MS, the anthocyanin compounds of cyanidin 3-rutinoside ([M + H]^+^ at *m*/*z* 595) and peonidin 3-rutinoside ([M + H]^+^ at *m*/*z* 609) in *P. spinosa* fruits, and highlighted that the colorant capacity of these fruits is mainly attributed to these compounds. Although authors quantify the content of those anthocyanin compounds in *P. spinosa* fruits, the conditions of extraction were not optimized. Therefore, based on those preliminary findings, it seems logical to continue to explore the potential of *P. spinosa* fruits as a source of anthocyanin compounds. In this regard, the study applies a RSM technique under a *CCCD* to optimize the operating conditions of the extraction of two common techniques in the industrial environment (HAE and UAE) with the intention of maximizing their extraction. However, because the major quantity of anthocyanin compounds in *P. spinosa* fruits is located in the fruit epicarp, in this study, we ignored the inside parts of the fruit and focused the attention on the fruit epicarps. Additionally, by focusing on the epicarps, we are avoiding a high content of interfering compounds in the extraction process (e.g., sugars) that would require further purification steps. Figure S1 (supplemental material) shows a complete summary of all the steps used for the optimization procedure in order to recover the anthocyanin compounds from the epicarps of *P. spinosa* fruits. Figure S2 (supplementary material) shows a chromatographic example of HPLC-DAD-ESI/MS results for the quantification of anthocyanin compounds in the epicarps of *P. spinosa* fruits.

Table 1 shows the experimental results derived from the *CCCD* used to optimize the extraction of anthocyanins from the fruits epicarps (*Y_1_*, mg C/g R; *Y_2_*, mg C/g E dw; and *Yield,* %) for each one of the computed extraction techniques (HAE and UAE). As described, the *CCCD* experimental results are subjected to the mathematical analysis of Equation (1), by applying a fitting procedure coupled with non-linear least-squares estimations. The parametric values of Equation (1) derived from this analytical procedure, the corresponding confidence interval of the parameters (α = 0.05) found after modelling the extraction response values, and basic statistical information of the mathematical procedure are presented in Table 2. The parametric values considered non-significant (*ns*) values were excluded from model construction and the final equations for describing the responses assessed using significant terms are presented in Table S1 (supplementary material).

The significant parametric values in Table 2 are presented as a function of the codification criteria of the *CCCD*. Although they could be presented as the real numerical ranges of the variables assessed (*X_1_* to *X_3_*), such information would not provide any additional insights of the regression analysis performed or the possible effects that may occur. The key information is the weight of the numerical values of the significant parameters; therefore, it seems logical to present them under a codification mode that allows us to compare the values between them effortlessly. Therefore, based on the numerical values derived, some global conclusions can be deduced as follows:

- For the HAE technique: In global terms, the significant parametric values within the linear effect (LE) group have a far more relevant contribution to the description of the responses than the interactive effect (IE), with the quadratic effect (QE) group being the less representative one (LE > IE >> QE). In the extraction *Yield* response, the three variables assessed (*t*, *T*, and *S*) showed similar contributions to its description. Regarding the response values of *Y_1_* (mg CT/g R) and *Y_2_* (mg CT/g E dw), the contribution of the variables is *S* >> *T* > *t*.
- For the UAE technique: The contribution to the description of effects of the responses by the significant parametric values is distributed as LE > QE >> IE. In all the responses assessed (extraction *yield*, *Y_1_* and *Y_2_* values), the contribution of the variables is *S* >> *P* > *t*.

In general, positive and highly significant effects of LE, QE, and IE are found to moderately affect the studied responses. In both techniques assessed (HAE and UAE), the variable *S* is the most relevant one. Initial increases of *S* cause an increase of the extraction efficiency until it reaches a maximum, in which case the increase will cause a decrease in the extraction, but its interactive effect with the variable *t* and *T* or *P* causes a more favorable influence.

Additionally, using the significant parametric values of Table 2 coupled with a simplex methodology, it is possible to determine the absolute/relative optimal values of conditions to maximize the responses individually or globally, in order to obtain the most efficient extraction process. Table 3 shows the individual and global optimal response values and the corresponding conditions that produced them. In consequence, the extracting techniques (HAE and UAE) according to the three response value formats (*Y_1_*, mg C/g R; *Y_2_*, mg C/g E dw; and *Yield,* %) for each assessed anthocyanin (C1 and C2), as well as for the total anthocyanin content (CT = C1 + C2), are depicted.

### 2.2. Alternative Visual Illustration of the Effects of the Extraction Variables on the Target Responses Used

**Figure 1:**
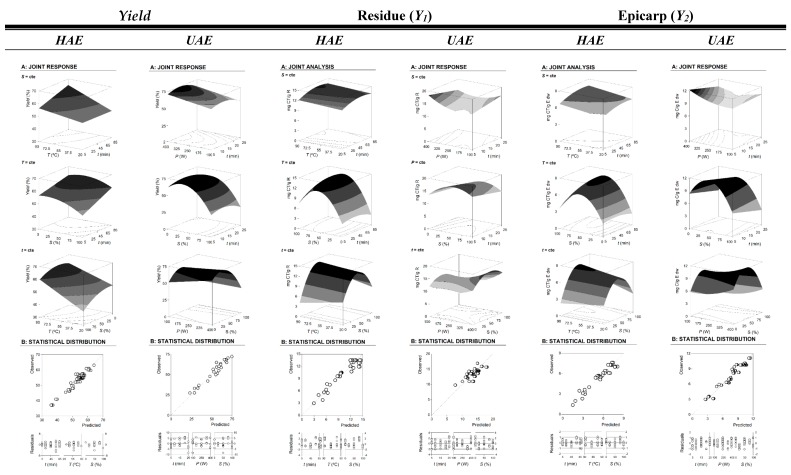
Illustration of the graphical results obtained by heat assisted extraction (HAE) and ultrasound assisted extraction (UAE) for the extraction *yield* of the residual content material (R) and the total detected anthocyanin compounds (cyanidin 3-rutinoside and peonidin 3-rutinoside, CT = C1 + C2) in terms of two response formats (*Y_1_*, mg C/g R and *Y_2_*, mg C/g E dw). Full results are described in Table 1. Every figure is presented in two parts. Part A shows the 3D net surfaces predicted by Equation (1) when the excluded variable is positioned at the individual optimum (Table 3). Part B describes the statistical analysis in a graphical form to show the goodness of fit of the models applied.

**Figure 2:**
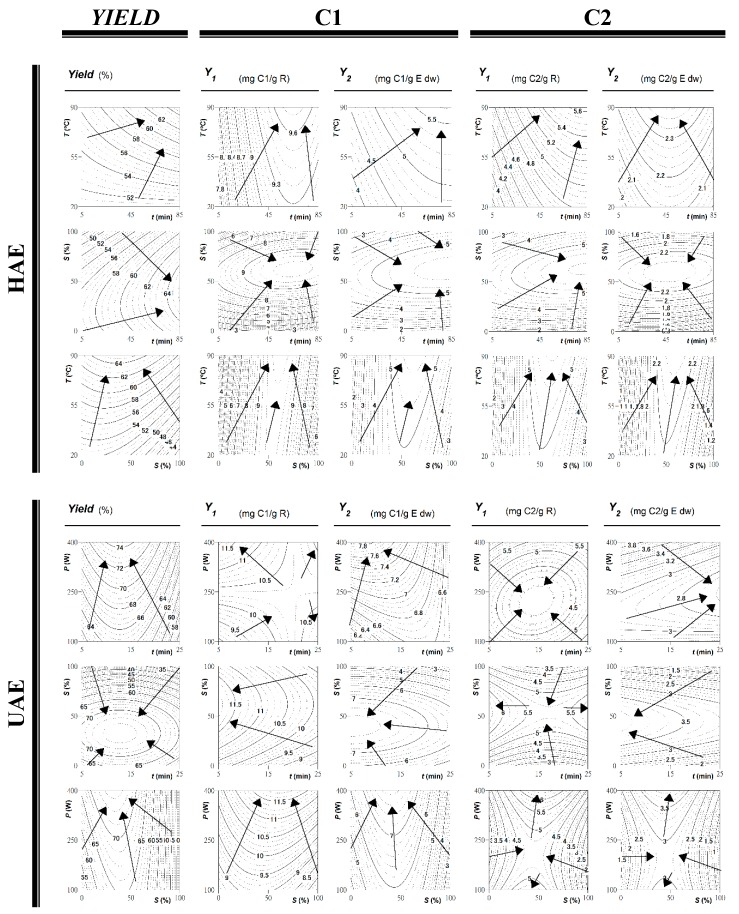
The optimized isolines projections for the extraction of C1 (cyanidin 3-rutinoside) and C2 (peonidin 3-rutinoside) as a function of the combination of the three main variables involved (*X_1_*, *X_2_*, and *X_3_*) in the HAE and UAE. For each compound, the two response value formats (*Y_1_*, mg C/g R and *Y_2_*, mg C/g E dw) are presented to describe the most favorable conditions. Furthermore, the response projections of the *yield* of the extracted residual material are presented. All the contour graphs were built by the second order polynomial models generated by Equation (1) (Table S1) when the excluded variable is positioned at the individual optimum (Table 3).

Although the parametric values show the behavior of the responses, and could be used to understand their patterns, a more visual way to express the effects of variables on the extraction of any type of response is to generate 3D surface and/or contour plots, by varying two variables in the experimental range under investigation and holding the other one at a fixed level. In this regard, Figure 1 and Figure 2 show the 3D surface and 2D contour plots, respectively, representing the influence of the investigated effects of HAE and UAE parameters on the extraction behavior. The plots enable one to visualize the influence and interaction between the variables. Visual analyses of 3D surface and 2D contour plots are in accordance with parametric values derived from the multiple regression analysis, as described in Table 2 (parametric values) and Table S1 (full mathematical models, supplementary material).

The extraction results for HAE and UAE, as function of the combination of the three main involved variables (*X_1-3_*: *t*, *T* or *P*, and *S*), can be observed in Figure 1 and Figure 2. In this regard, Figure 1 shows the 3D surface plots of the extracted R (*Yield*, %), and CT, in two response formats (*Y_1_*, mg CT/g R and *Y_2_*, mg CT/g E dw). On the other hand, Figure 2 shows the optimized isolines projections for C1 (cyanidin 3-rutinoside) and C2 (peonidin 3-rutinoside) extraction, in the two response value formats (*Y_1_*, mg C/g R and *Y_2_*, mg C/g E dw). These figures show, respectively, optimized 3D graphical and 2D isolines projection results for the extracted anthocyanins (C1 or C2) as function of the three combined variables (*t*, *T* or *P*, and *S*) in HAE and UAE. The total anthocyanins (C1+C2) are accounted together (CT) in Figure 1, and individually in Figure 2. They are helpful to visualize the tendencies of each response and lead to define of the maximum favorable conditions, considering all together all responses.

Additionally, Figure 1B exemplifies the competence to predict the obtained results. In statistical terms, the distribution of residues (Figure 1) presents, for the majority of the cases, more than 90% of reliability, showing a good agreement between experimental and predicted values. This is also verified by the achieved high *R^2^* values (Table 2), which indicates the percentage of variability explained by the model.

In HAE, small differences between the extraction behavior of the two considered anthocyanins (when comparing C1 and C2, or *Y_1_* and *Y_2_*) were clearly distinguished. The opposite occurred in UAE, the effects were distinct for each one of the detected anthocyanins, as well as according to the response format. However, for both extraction techniques, the *S* variable was the most significant one, producing a relevant impact on the level of extraction of all anthocyanins assessed. As described above, the LE and the QE of the significant parametric values of the variable *S* can be perceived in all figures. In almost all cases, the variable *S* indicates a maximum level at ~50% of hydroalcoholic mixture (water/ethanol, *v*/*v*). The negative impact of quadratic term of the variable *S* can be explained through the increase of water in the process, which expands the yield of extraction. Other negative effects such as those between *T* or *P* and *S* may suggest that the further use of lower *P*, in combination with higher *S*, will avoid the anthocyanin degradation. The results are in accordance with others recently reported by other authors [19,20,21], in which inclined surfaces to the side of *T* or *P* may increase the solubility of target compounds by using stronger energies, and consequently improve their release from the sample matrix, while destroying the integrity of connective and structural tissues. 

### 2.3. Conditions That Maximize the Anthocyanins Extraction and Experimental Verification 

**Figure 3:**
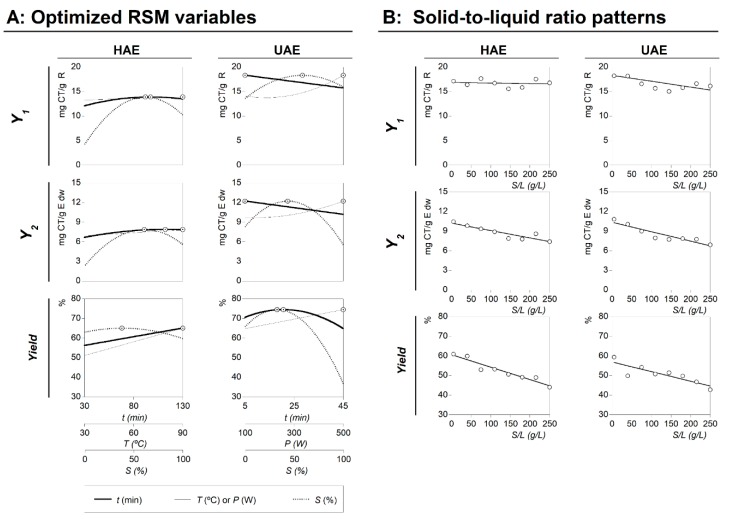
Final graphical effects of all variables assessed for HAE and UAE systems. Part A shows the individual 2D responses as a function of all the variables assessed that were positioned at the individual optimal values of the others (Table 2). The points (⊙) presented alongside each line highlight the location of the optimum value. Lines and dots are generated by the theoretical second order polynomial models generated by Equation (1) (Table S1). Part B shows the dose response of *S/L* at the global optimal values of the other three variables (Table 3). The limit value (~150 g/L) shows the maximum achievable experimental concentration until the sample cannot be physically stirred at laboratory scale. RSM—response surface methodology.

The aim of this study was to maximize the extraction yield of targeted anthocyanin compounds from epicarps of *P. spinosa* fruits, in the applied HAE and UAE techniques, within pre-set variable conditions and ranges. The values of the variable conditions that lead to optimal response values for RSM using a *CCCD*, obtained with the aid of *simplex* procedure, for each of the assessed extracting techniques are shown in Table 3. Figure 3 part A shows the individual summary of the effects of all variables assessed for HAE and UAE systems in 2D illustrations, where the variables are positioned at the individual optimal values of the others (Table 3). The dots (⊙) presented alongside each line highlight the location of the optimum value, meanwhile lines are the predicted behavior found by the mathematical analysis of Equation (1) generated by the theoretical second-order polynomial models described in Table S1. Next, some relevant details of the results produced are highlighted:

- For the HAE: the global optimal variable conditions were found at 49.02 ± 2.94 min, 90.00 ± 7.20 °C, and 50.00% ± 0.50% of ethanol, producing maximum response values of 13.93 ± 0.42 mg CT/g R (*Y_1_*), 7.93 ± 0.08 mg CT/g E dw (*Y_2_*), and 50.89% ± 3.05% (*yield* of the extracted residue).
- For the UAE: the global optimal variable conditions were found at 5.00 ± 0.15 min, 400.00 ± 32.00 W, and 47.98% ± 2.88% of ethanol, producing maximum response values of 18.17 ± 1.82 mg CT/g R (*Y_1_*), 11.76 ± 0.82 mg CT/g E dw (*Y_2_*), and 68.60% ± 2.06 % (*yield* of the extracted residue).

Considering both the individual and global values, the higher amount of extracted compounds was obtained for the UAE technique. The ideal solvent composition was almost the same, and the two techniques required high energy values, where the highest values of *T* and *P* proposed by the experimental design were the optimal, but the UAE needed less *t* than HAE (~90% less). The obtained results are in accordance with similar conclusions found previously [10,17,22], in which UAE proved to consume less energy because of the lower *t* needed, and provide higher extraction values while increasing the purity and, additionally, aiding to meet the requirements of a green extraction concept.

### 2.4. Dose-Response Analysis of the Solid-to-Liquid Ratio Effect at the Optimal Conditions

The study of *S*/*L* effect was performed at the optimal conditions (Table 3) predicted by the polynomial models obtained for each extraction technique (HAE and UAE) using the total anthocyanin content (CT), as quantified by HPLC analysis, as the response factor. The individual *S*/*L* study for each individual anthocyanin (C1 or C2) was not presented because the behavior was similar to the pattern of the total amount. In both processes, the *S*/*L* was designed to verify the behavior between 5 and 250 g/L. The maximum value of 250 g/L was used as a limit condition because of the impossibility of producing a homogenized extraction when higher values were used. 

The obtained dose responses of the *S*/*L* were consistent for both HAE and UAE systems, and could be described by a simple linear relationship (shown in Part B of Figure 3). All experimental points are distributed around the linear equation; consequently, the dose response is explained by the slope (*m*) of the linear relationship. None of the cases showed positive *m* values (the extraction efficiency increases as the *S/L* rate increases), and two cases showed non-significant values or a zero value of *m* (the efficiency doesn’t change as the *S*/*L* increases). In all the other cases, the *m* showed negative values (the efficiency decreases as the *S*/*L* increases). The responses from the *Y_1_* value format, for HAE and UAE, were the ones that showed non-significant *m* values, whereas all other responses showed significant negative values of *m* (*Y_1_* and *Yield* for HAE and UAE). The conclusions derived from this analysis are described below:

- For the *Y_1_* value format, the response of the parametric *m* value in HAE and UAE presents a non-significant interval of confidence, which means that the changes in the response are not statistically supported and, therefore, the parameter must be considered equal to zero. In other words, the amount of anthocyanins in the extracted residue does not vary as a function of the *S/L* increase. The extraction values were defined numerically by the intercept parametric value (*b*) of the linear equation as 14.85 ± 2.29 and 18.25 ± 3.95 mg CT/g R for HAE (*R^2^* = 0.9920) and UAE (*R^2^* = 0.9817), respectively.
- For the *Y_2_* value format, the parametric values for HAE were *b* = 9.21 ± 1.37 mg CT/g E dw and *m* = −0.0113 ± 0.0051, with *R^2^* = 0.9566; while for UAE, *b* = 10.32 ± 1.48 mg CT/g E dw and *m* = −0.0143 ± 0.0038, with *R^2^* = 0.9244. Negative *m* values show that the *S/L* increase leads to a decrease in the extraction ability, obtaining a maximum value of extraction at 5 g/L and a minimum at 250 g/L. However, the observed decrease is slight (less than −0.02), which means that the increase of 1 g/L implies the loss of 0.0113 ± 0.0051 mg CT/g E dw for the HAE process and 0.0143 ± 0.0038 mg CT/g E dw for UAE. Such values produce losses at the maximum tested experimental value (250 g/L) of ~15%, comparative with the one extracted at 5 g/L. Nevertheless, the economic advantages of working at 250 g/L are far more superior than the possible benefits of extracting at the optimal *S/L* value.
- For the *Yield* value format, the parametric values for HAE were *b* = 54.62% ± 4.87% and *m* = −0.0636 ± 0.0123, with *R^2^* = 0.9516; whereas for UAE, *b* = 58.90% ± 7.77% and *m* = −0.0491 ± 0.0116, with *R^2^* = 0.9618. Although, at the initial *S/L* values, the results obtained for HAE and UAE conducted to similar extraction yields, these values decreased as the *S*/*L* increased. The *m* parametric value is significantly lower for the UAE process, resulting in higher extraction yield values at 250 g/L. These results are in accordance with the conclusions highlighted in the literature, where UAE is reported as enhancing the extraction process by increasing the mass transfer between the plant material and the solvent [23]. The UAE leads to better cell disruption, facilitating the release of the extractable compounds by increasing the contact surface area between the solid and liquid phases [22,23].

### 2.5. Comparison with Other Studies Involving the Extraction of Anthocyanins

There are few works in the literature dealing with anthocyanins in *P. spinosa* fruits. In one of these studies with *P. spinosa* fruits, Guimarães et al. [5] performed the extraction using methanol with 0.5% TFA added as solvent, and identified eight different anthocyanins, predominantly peonidin 3-rutinoside and cyanidin 3-rutinoside, with 34.47 ± 0.03 µg/100 g fruits dw and 31.12 ± 0.11 µg/100 g fruits dw, respectively. Other authors [6], found 3.5 ± 0.5 mg of anthocyanins/100 g dw of *P. spinosa* fruits. Both authors used the whole fruit, while in this study, only the epicarp was used as the extraction material, a fact that may justify the significant differences between the encountered results, when compared with the present study. Compared with the pulp, the fruit epicarp presents a greater intensity of color and, therefore, a higher concentration of anthocyanins, in addition to less interfering compounds, is obtained. Moreover, another fact that aided the production of large amounts of anthocyanins from the extracted material was the optimization of the extraction process, which led to increased extraction efficiency and yield. In another study that used *P. spinosa* fruits as a source of anthocyanins [6], the total content was quantified by spectrophotometric methods, presenting values that cannot be compared with those found in the present study. 

Some examples of other plant-based sources of anthocyanins are *Oryza sativa* L. (var. Glutinosa) bran, which shows 42.00 mg/g [24]; *Phaseolus vulgaris* L. (common beans) fruit coat, presenting 32.00 mg/g [25]; and *Rubus fruticosus* L. (blackberries) fruit, which possess 17.10 mg/g [26]. Although these values are slightly higher than those presented by *P. spinosa* fruits, in general, the referred fruits and vegetables already have a high commercial value and other industrial purposes, unlike *P. spinosa* fruits. On the other hand, residues such as grape vine (*Vitis vinifera* L.) pomace, and mango (*Mangifera indica*) skin presented lower anthocyanin amounts, that is, 6.33 mg/g [27] and 2.03 to 3.60 mg/g [28], respectively. Thus, these wild fruits revealed to be an excellent source of anthocyanins, serving as a base raw-material for the production of natural colorant additives for commercial purposes.

## 3. Discussion

The minimalism of using conventional methods (HAE or maceration) versus the compensations of new non-conventional technologies (microwave, ultrasound, cold pressing, squeezing, etc.) to recover compounds from plant materials, as well as by-products, is a principal topic in the list of many industries in order to increase profitability by decreasing energy costs and reducing greenhouse gas emissions to meet legal requirements. Additionally, non-conventional technologies favor the safety of processes and the quality of products, as well as the functionality and product standardization.

Scientific literature shows clear evidence that extraction procedures of target compounds from plant-based products must be assessed individually. Therefore, a nonstop effort needs to be performed, as agro-industrial and food sectors are looking for by-products’ valorization into added-value products. However, in order to take full advantage of the technological advances, the extraction conditions need to be optimized. Mathematical solutions, such as RSM tools, could increase the efficiency and profitability of the process and help to change conventional extraction approaches. 

Colorants are one of the most important additives in terms of marketing because their presence in food products is considered to influence customers’ perceptions, choices, and preferences. *P. spinosa* fruit epicarps have been scarcely explored and, to the best of the authors knowledge, the potential industrial use of their anthocyanin compounds has not been previously investigated. In such a context, the present work presents a new rapid method to extract anthocyanin compounds from *P. spinosa* fruit epicarps. RSM and other mathematical strategies were successfully employed to optimize the extraction conditions that maximize the anthocyanin compounds’ recovery to produce a rich extract with potential industrial application as a natural coloring additive. 

## 4. Materials and Methods 

### 4.1. Plant Material

Ripe *P. spinosa* fruits were harvested in Bragança (Trás-os-Montes, Northeast Portugal) in September 2017, the epicarp was separated from the rest of the fruit body, frozen, and lyophilized. They were then triturated, to be reduced to a fine powder (~20 mesh), and stored under refrigeration, protected from light until further use.

### 4.2. Extraction Procedures for P. Spinosa Fruit Epicarps

#### 4.2.1. Heat Assisted Extraction (HAE)

HAE was performed in a water reactor agitated internally with a Cimarec^TM^ Magnetic Stirrer at a constant speed (~500 rpm, Thermo Scientific, San Jose, CA, USA), following a procedure previously performed [13]. The powdered epicarp samples of *P. spinosa* (1.0 g) were extracted with 20 mL of solvent (ethanol/water) acidified with citric acid (pH = 3), under diverse conditions, as previously defined by the established RSM plan (Table 1). The ranges of the experimental design were as follows: time (*t* or *X_1_,* 5 to 85 min), temperature (*T* or *X_2_,* 20 to 90 °C), and ethanol content (*S* or *X_3_,* 0% to 100%). The solid-to-liquid ratio (*S/L* or *X_4_*) was kept at 50 g/L for all conditions.

#### 4.2.2. Ultrasound-Assisted Extraction (UAE)

An ultrasonic device (QSonica sonicators, model CL-334, Newtown, CT, USA) equipped with a water reactor (EUP540A, Euinstruments, France) at a fixed frequency (40 kHz) was used for UAE procedure. The variables considered were as follows: ultrasonic power (*P*, in watts), *S*, and *t*, which were programmed according to the defined RSM plan (Table 1), following a procedure previously performed [29]. The powdered epicarp samples (2.5 g) were placed in a reactor with 50 mL of solvent (ethanol/water) acidified with citric acid (pH = 3), and extracted under diverse conditions, maintaining the *S/L* constant at 50 g/L. The ranges of the experimental design were as follows: *t* (*X_1_*, 5 to 25 min), *P* (or *X_2_*, 100 to 400 W), and *S* (or *X_3_*, 0% to 100%).

#### 4.2.3. Post-Extraction Sample Processing

When all the individual extraction conditions were carried out (for HAE and UAE), the samples were immediately centrifuged (6000 rpm during 20 min at 10 °C) and filtered (paper filter Whatman nº 4) to eliminate the non-dissolved material. The supernatant was collected and divided into two portions for HPLC and extraction yield analysis. The portion separated for HPLC analysis (3 mL) was dried at 35 °C, re-dissolved in acidified water (citric acid solution with pH 3), and filtered through an LC filter disk (0.22 µm), whereas the portion for the extraction yield determination (5 mL) was dried at 105 °C during 48 h and thereafter weighted.

### 4.3. Identification and Quantification of Anthocyanins by HPLC

The extract was analyzed using an HPLC-DAD-ESI/MSn (Dionex Ultimate 3000 UPLC, Thermo Scientific, San Jose, CA, USA) system, previously described [30]. The detection was carried out using a DAD (520 nm as the preferred wavelength) and mass spectrometer (Linear Ion Trap LTQ XL mass spectrometer, Thermo Finnigan, San Jose, CA, USA) equipped with an ESI source. The anthocyanins present in the samples were characterized according to their UV and mass spectra. The anthocyanins cyanidin 3-rutinoside (C1) and peonidin 3-rutinoside (C2) were the most relevant compounds found, and were, therefore, quantified using a five-level calibration curve of known concentrations (200–20 µg/mL) of cyanidin 3-glucoside (*y* = 243287 *x* − 1000000; *R^2^* = 0.9953, Polyphenols, Sandnes, Norway) and peonidin 3-glucoside (*y* = 122417 *x* − 447974; *R^2^* = 0.9965, Polyphenols, Sandnes, Norway).

### 4.4. Response Value Formats for Results Presentation

The two anthocyanin compounds (C, either C1 or C2) and their sum (C total, CT) were used as responses in each applied technique. The results were presented according to two response formats (*Y*): *Y_1_*, in mg of C per gram of extracted residue (mg C/g R), which was specifically used to evaluate the C purity in the extracts; and *Y_2_*, in mg of C per g of fruit epicarp dry weight (mg C/g E dw), which was specifically used to analyses the C extraction yield. Both responses were equally analyzed, but additional considerations regarding the last one (*Y_2_*, mg C/g E dw) were taken in the results presentation, becuse it is considered as an important guiding response when dealing in terms of optimization for industrial transference. Note that by dividing those responses, *Y_2_*/*Y_1_,* the extracted residue quantity (g R/g E dw) is obtained, which provides information regarding the third response criterion expressed (*Yield*, %).

### 4.5. Experimental Design, Model Analysis, and Statistical Evaluation

#### 4.5.1. RSM Experimental Design

Trials based on one-at-the-time analysis (analysis of each of the variables for each one of the selected techniques) were conducted, the ranges originating significant changes were selected (Table 1). The joint effects of the three defined variables were studied using a *circumscribed central composite design* (*CCCD*), using five levels for each one with twenty eight response combinations, as described previously [31].

#### 4.5.2. Mathematical Model

The experimental data produced by the RSM design were analyzed mathematically by means of least-squares calculation, using the following second-order polynomial equation with interactive terms [32]:

(1) $$Y=b_{0}+\sum_{i=1}^{n}b_{i}X_{i}+\sum_{\begin{array}{l} i=1\\ j>i \end{array}}^{n-1}\sum_{j=2}^{n}b_{ij}X_{i}X_{j}+\sum_{i=1}^{n}b_{ii}X_{i}^{2}$$

where *Y* is the dependent variable (response variable) modelled, *X_i_* and *X_j_* define the independent variables, *b_0_* is the constant coefficient, *b_i_* is the coefficient of linear effect, *b_ij_* is the coefficient of interaction effect, *b_ii_* the coefficient of quadratic effect, and *n* is the number of variables. As responses, the following three value formats were used: *Y_1_* (mg C/g R), *Y_2_* (C/g E dw), and *Yield* (%).

#### 4.5.3. Procedure to Optimize the Variables to a Maximum Response

A simplex algorithm method was used to find the optimum values by solving nonlinear problems in order to maximize the extraction yield and the recovery of anthocyanin compounds, as explained previously [33]. Certain limitations were imposed (i.e., times cannot be lower than 0) to avoid variables with unnatural and unrealistic physical conditions.

#### 4.5.4. Dose-Response Analysis of the Solid-to-Liquid Ratio 

Once the optimal conditions (*X_1_*, *X_2_*, and *X_3_*) were found, the following natural optimization step was used to describe the pattern of the *S*/*L* (or *X_4_*, expressed in g/L). The objective was to achieve more productive conditions as required by industrial applications. In all cases, experimental points are distributed following linear patterns as the *S*/*L* increases, consequently, linear models with intercepts were used to evaluate the responses. The parametric value of the slope (*m*) was used to assess the dose response. Positive values will indicate an increase in the extraction responses, whereas negative values will designate a decrease in the extraction efficiency, as the *S*/*L* increases. 

### 4.6. Mathematical Procedures

The analytical procedures to model the data, to determine the parametric values, confidence intervals, and statistical calculations, were obtained following the descriptions of other authors [34]. In brief, (a) the parametric values were obtained using the quasi-Newton algorithm (least-square) by running the integrated macro ‘*Solver*’ in Microsoft Excel; (b) the coefficient significance of the parameters produced (α = 0.05) was assessed using the ‘*SolverAid*’ macro to conclude their confidence intervals; and c) the model consistency was proven by means of several statistical criteria, such as (i) the Fisher *F*-test (α = 0.05); (ii) the ‘*SolverStat*’ macro; and (iii) the *R²* coefficient.

## 5. Conclusions

The efficiency of the UAE was higher than that obtained with HAE. The main anthocyanins identified were cyanidin 3-rutinoside and peonidin 3-rutinoside, being the ones quantified. Through the optimization of the extraction process, it was possible to reach by UAE 18.17 ± 1.82 mg CT/g R (*Y_1_*), 11.76 ± 0.82 mg CT/g E dw (*Y_2_*), and 68.60% ± 2.06% (yield of the extracted residue), with the optimal parameters of extraction being 5.00 ± 0.15 min, 400.00 ± 32.00 W, and 47.98% ± 2.88% of ethanol. The used mathematical models (RSM and dose-response models) were statistically significant and allowed the optimization of the anthocyanins extraction. For the *S*/*L* effects, inspected at the optimum conditions, the responses for all assessed criteria followed a decreasing linear relation until 250 g/L.

In conclusion, the present study contributed to the valorization of the wild fruits of *P. spinosa* by exploring anthocyanin-rich extracts that can find potential application as natural colorants in different industrial fields. For that purpose, an optimized extraction method was obtained using advanced and efficient extraction systems.

## Figures and Tables

**Table 1:** Experimental results of the *CCCD* used for the response surface methodology (RSM) optimization of the three main variables involved (*X_1_*, *X_2_*, and *X_3_*) in the heat assisted extraction (HAE) and ultrasound assisted extraction (UAE). Responses comprised three format values assessed (*Y_1_*, mg C/g R; *Y_2_*, mg C/g E dw; and *Yield*, %).

Experimental Design										HAE							UAE						
	Coded Values			HAE			UAE			Residue	Individual Content				Total Content		Residue	Individual Content				Total Content	
	*X_1_*	*X_2_*	*X_3_*	*X_1_*: *t*	*X_2_*: *T*	*X_3_*: *S*	*X_1_*: *t*	*X_2_*: *P*	*X_3_*: *S*	*Yield*	*Y_1_*C1	*Y_1_*C2	*Y_2_*C1	*Y_2_*C2	*Y_1_*CT	*Y_2_*CT	*Yield*	*Y_1_*C1	*Y_1_*C2	*Y_2_*C1	*Y_2_*C2	*Y_1_*CT	*Y_2_*CT
				min	°C	%	min	W	%	%	mg/g R	mg/g R	mg/g E	mg/g E	mg/g R	mg/g E	%	mg/g R	mg/g R	mg/g E	mg/g E	mg/g R	mg/g E
**1**	−1	−1	−1	21.2	34.2	20.3	9.1	160.8	20.3	53.75	6.65	3.04	3.58	1.64	9.73	5.21	61.24	9.26	3.65	5.67	2.24	12.91	7.91
**2**	−1	−1	1	21.2	34.2	79.7	9.1	160.8	79.7	45.43	7.54	4.65	3.42	2.11	12.22	5.54	52.87	9.45	4.44	5.00	2.35	13.89	7.34
**3**	−1	1	−1	21.2	75.8	20.3	9.1	339.2	20.3	57.26	6.08	2.81	3.48	1.61	8.78	5.09	70.17	8.96	3.44	6.29	2.41	12.40	8.70
**4**	−1	1	1	21.2	75.8	79.7	9.1	339.2	79.7	48.85	8.35	4.44	4.08	2.17	12.74	6.25	58.80	9.36	4.51	5.50	2.65	13.87	8.15
**5**	1	−1	−1	68.8	34.2	20.3	20.9	160.8	20.3	55.45	6.88	2.90	3.82	1.61	9.66	5.42	59.12	10.66	3.69	6.30	2.18	14.35	8.48
**6**	1	−1	1	68.8	34.2	79.7	20.9	160.8	79.7	50.00	10.05	4.05	5.03	2.02	14.03	7.05	55.22	8.99	4.14	4.97	2.29	13.14	7.25
**7**	1	1	−1	68.8	75.8	20.3	20.9	339.2	20.3	60.18	6.72	2.81	4.04	1.69	9.37	5.73	67.05	8.79	2.86	5.90	1.92	11.65	7.82
**8**	1	1	1	68.8	75.8	79.7	20.9	339.2	79.7	53.09	11.01	4.39	5.84	2.33	15.26	8.17	57.19	8.69	4.24	4.97	2.42	12.92	7.39
**9**	−1.68	0	0	5	55	50	5	250	50	53.88	8.15	3.80	4.39	2.05	12.01	6.44	68.58	10.53	4.77	7.22	3.27	15.30	10.49
**10**	1.68	0	0	85	55	50	25	250	50	56.51	9.09	3.70	5.14	2.09	12.74	7.23	56.13	10.14	4.56	5.69	2.56	14.71	8.25
**11**	0	−1.68	0	45	20	50	15	100	50	49.49	11.09	4.70	5.49	2.33	15.59	7.82	55.99	12.41	5.45	6.95	3.05	17.86	10.00
**12**	0	1.68	0	45	90	50	15	400	50	60.78	8.68	3.42	5.27	2.08	11.96	7.36	76.95	10.60	4.40	8.16	3.38	15.00	11.54
**13**	0	0	−1.68	45	55	0	15	250	0	54.73	3.81	1.63	2.09	0.89	5.46	2.98	50.18	8.22	1.92	4.12	1.19	10.14	5.31
**14**	0	0	1.68	45	55	100	15	250	100	47.62	5.68	2.69	2.70	1.28	8.23	3.99	34.40	11.06	1.15	3.81	0.39	12.21	4.20
**15**	−1.68	−1.68	−1.68	5	20	0	5	100	0	54.39	4.24	1.85	2.30	1.01	6.01	3.31	47.94	8.15	3.73	3.91	1.79	11.88	5.69
**16**	−1.68	−1.68	1.68	5	20	100	5	100	100	36.34	2.45	1.65	0.89	0.60	4.12	1.49	33.12	6.25	1.50	2.07	0.50	7.75	2.57
**17**	−1.68	1.68	−1.68	5	90	0	5	400	0	56.79	3.53	1.59	2.00	0.90	5.13	2.90	61.16	10.89	4.46	6.66	2.73	15.36	9.39
**18**	−1.68	1.68	1.68	5	90	100	5	400	100	47.24	4.51	2.39	2.13	1.13	6.88	3.26	28.92	11.57	4.64	3.34	1.34	16.21	4.69
**19**	1.68	−1.68	−1.68	85	20	0	25	100	0	51.72	4.45	2.00	2.30	1.03	6.40	3.33	45.30	9.99	4.55	4.53	2.06	14.54	6.59
**20**	1.68	−1.68	1.68	85	20	100	25	100	100	39.88	4.46	1.75	1.78	0.70	6.21	2.48	28.91	9.60	1.71	2.78	0.50	11.31	3.27
**21**	1.68	1.68	−1.68	85	90	0	25	400	0	64.04	2.14	0.78	1.37	0.50	2.95	1.87	51.96	9.85	3.88	5.12	2.02	13.73	7.13
**22**	1.68	1.68	1.68	85	90	100	25	400	100	61.84	6.75	2.82	4.17	1.74	9.54	5.91	23.34	10.08	3.72	2.35	0.87	13.80	3.22
**23**	0	0	0	45	55	50	15	250	50	56.07	8.65	3.78	4.85	2.12	12.53	6.97	65.72	10.85	4.50	7.13	2.96	15.35	10.08
**24**	0	0	0	45	55	50	15	250	50	56.55	8.79	4.13	4.97	2.34	13.00	7.31	65.90	10.93	4.34	7.20	2.86	15.27	10.06
**25**	0	0	0	45	55	50	15	250	50	54.57	8.99	4.09	4.91	2.23	13.22	7.14	66.06	10.44	4.04	6.89	2.67	14.47	9.56
**26**	0	0	0	45	55	50	15	250	50	54.35	8.65	3.78	4.70	2.05	12.36	6.76	67.94	11.08	4.35	7.53	2.96	15.43	10.48
**27**	0	0	0	45	55	50	15	250	50	54.57	9.26	4.20	5.02	2.27	13.33	7.14	67.80	10.27	4.09	6.96	2.77	14.36	9.74
**28**	0	0	0	45	55	50	15	250	50	54.35	9.12	4.18	5.02	2.30	13.37	6.76	68.10	10.26	3.92	6.99	2.67	14.18	9.66

**Table 2:** Parametric results of the second-order polynomial equation of Equation (1) for the HAE and UAE techniques assessed and for the three response value formats (*Y_1_*, mg C/g R; *Y_2_*, mg C/g E dw; and *Yield,* %). The parametric subscript 1, 2, and 3 stands for the variables involving *t* (*X_1_*), *T* or *P* (*X_2_*), and *S* (*X_3_*), respectively. Analyses of significance of the parameters (α = 0.05) are presented in coded values. Additionally, the statistical information of the fitting procedure to the model is presented.

Parameters		Residue	Individual Content				Total Content	
		*Yield*	*Y_1_*C1	*Y_1_*C2	*Y_2_*C1	*Y_2_*C2	*Y_1_*CT	*Y_2_*CT
**HAE**								
Intercept	*b_0_*	54.86 ± 0.72	9.35 ± 0.38	4.15 ± 0.19	5.07 ± 0.17	2.26 ± 0.08	13.48 ± 0.55	7.29 ± 0.23
Linear effect	*b_1_*	1.54 ± 0.43	0.33 ± 0.21	ns	0.25 ± 0.09	ns	0.28 ± 0.21	0.26 ± 0.13
	*b_2_*	3.12 ± 0.43	ns	ns	0.15 ± 0.09	0.05 ± 0.02	ns	0.19 ± 0.13
	*b_3_*	−3.07 ± 0.43	0.56 ± 0.21	0.33 ± 0.11	0.17 ± 0.09	0.11 ± 0.05	0.88 ± 0.31	0.27 ± 0.13
Quadratic effect	*b_11_*	ns	−0.26 ± 0.21	−0.09 ± 0.07	−0.10 ± 0.05	−0.05 ± 0.03	−0.35 ± 0.26	−0.16 ± 0.15
	*b_22_*	ns	ns	ns	ns	ns	ns	ns
	*b_33_*	−1.24 ± 0.42	−1.60 ± 0.26	−0.69 ± 0.13	−0.94 ± 0.11	−0.40 ± 0.06	−2.26 ± 0.37	−1.33 ± 0.15
Interactive effect	*b_12_*	0.78 ± 0.31	0.00 ± 0.00	ns	ns	ns	ns	ns
	*b_13_*	0.54 ± 0.31	0.26 ± 0.15	0.04 ± 0.02	0.18 ± 0.07	0.04 ± 0.03	0.31 ± 0.22	0.22 ± 0.09
	*b_23_*	0.69 ± 0.31	0.31 ± 0.15	0.14 ± 0.08	0.21 ± 0.07	0.09 ± 0.03	0.45 ± 0.22	0.29 ± 0.09
Statistics (*R²*)		0.9375	0.9100	0.8755	0.9443	0.9272	0.9046	0.9489
**UAE**								
Intercept	*b_0_*	68.11 ± 1.70	10.42 ± 0.47	4.10 ± 0.28	6.98 ± 0.22	2.83 ± 0.13	14.46 ± 0.62	9.75 ± 0.31
Linear effect	*b_1_*	−1.70 ± 0.96	ns	ns	−0.13 ± 0.12	−0.10 ± 0.07	ns	−0.22 ± 0.17
	*b_2_*	2.12 ± 0.96	0.23 ± 0.21	0.18 ± 0.15	0.25 ± 0.12	0.14 ± 0.07	0.37 ± 0.35	0.36 ± 0.17
	*b_3_*	−6.46 ± 0.96	ns	−0.16 ± 0.15	−0.55 ± 0.12	−0.26 ± 0.07	ns	−0.82 ± 0.17
Quadratic effect	*b_11_*	−2.29 ± 1.16	ns	0.26 ± 0.20	−0.13 ± 0.11	ns	ns	ns
	*b_22_*	ns	ns	0.35 ± 0.20	ns	0.19 ± 0.09	0.66 ± 0.42	0.27 ± 0.21
	*b_33_*	−7.33 ± 1.16	−0.36 ± 0.27	−0.84 ± 0.20	−1.02 ± 0.15	−0.68 ± 0.09	−1.22 ± 0.42	−1.84 ± 0.21
Interactive effect	*b_12_*	ns	−0.34 ± 0.20	−0.11 ± 0.11	−0.18 ± 0.09	−0.07 ± 0.05	−0.46 ± 0.25	−0.23 ± 0.12
	*b_13_*	ns	ns	ns	ns	ns	ns	ns
	*b_23_*	−1.29 ± 0.69	0.17 ± 0.10	0.22 ± 0.11	−0.08 ± 0.05	ns	0.41 ± 0.25	ns
Statistics (*R²*)		0.9431	0.7825	0.9032	0.9316	0.9035	0.8986	0.9380

**Table 3:** Variable conditions in natural values that lead to optimal global and individual response values for RSM for each of the extracting techniques assessed (HAE and UAE), for the three response value formats (*Y_1_*, mg C/g R; *Y_2_*, mg C/g E dw; and *Yield,* %), for each compound assessed (C1 and C2), and for the total compounds (CT = C1 + C2).

Criteria			Optimal Variable Conditions			Optimum Response	
			X_1_: t (min)	X_2_: T (°C) or P(W)	X_3_: S (%)		
**(A) Individual optimal variable conditions**							
HAE	*Yield*		85.00 ± 8.50	90.00 ± 4.50	38.01 ± 3.04	65.10 ± 3.91	%
	*Y_1_*	C1	64.89 ± 5.19	90.00 ± 8.10	62.01 ± 3.10	9.71 ± 0.49	mg C1/g R
		C2	47.18 ± 3.30	90.00 ± 7.20	62.22 ± 6.22	4.27 ± 0.34	mg C2/g R
		CT	58.85 ± 5.89	90.00 ± 9.00	61.97 ± 6.20	13.89 ± 0.14	mg CT/g R
	*Y_2_*	C1	84.27 ± 0.84	90.00 ± 3.60	63.11 ± 3.79	5.64 ± 0.51	mg C1/g E dw
		C2	48.06 ± 1.92	90.00 ± 0.90	59.98 ± 3.60	2.38 ± 0.21	mg C2/g E dw
		CT	70.76 ± 5.66	90.00 ± 4.50	60.82 ± 3.04	7.89 ± 0.55	mg CT/g E dw
UAE	*Yield*		12.79 ± 0.51	400.00 ± 32.00	32.51 ± 1.95	74.53 ± 2.24	%
	*Y_1_*	C1	5.00 ± 0.10	400.00 ± 28.00	61.80 ± 1.85	11.82 ± 0.71	mg C1/g R
		C2	5.00 ± 0.10	400.00 ± 28.00	53.58 ± 1.07	6.45 ± 0.45	mg C2/g R
		CT	5.00 ± 0.10	400.00 ± 20.00	58.39 ± 4.09	18.32 ± 1.47	mg CT/g R
	*Y_2_*	C1	5.00 ± 0.50	400.00 ± 4.00	40.11 ± 2.81	7.88 ± 0.16	mg C1/g E dw
		C2	5.00 ± 0.40	400.00 ± 28.00	44.35 ± 4.43	3.96 ± 0.28	mg C2/g E dw
		CT	5.00 ± 0.35	400.00 ± 20.00	43.37 ± 4.34	12.23 ± 0.86	mg CT/g E dw
**(B) Global optimal variable conditions**							
HAE	*Yield*		49.02 ± 2.94	90.00 ± 7.20	50.00 ± 0.50	50.89 ± 3.05	%
	*Y_1_*	C1				9.71 ± 0.29	mg C1/g R
		C2				4.22 ± 0.13	mg C2/g R
		CT				13.93 ± 0.42	mg CT/g R
	*Y_2_*	C1				5.57 ± 0.11	mg C1/g E dw
		C2				2.36 ± 0.05	mg C2/g E dw
		CT				7.93 ± 0.08	mg CT/g E dw
UAE	*Yield*		5.00 ± 0.15	400.00 ± 32.00	47.98 ± 2.88	68.60 ± 2.06	%
	*Y_1_*	C1				11.74 ± 0.23	mg C1/g R
		C2				6.43 ± 0.32	mg C2/g R
		CT				18.17 ± 1.82	mg CT/g R
	*Y_2_*	C1				7.81 ± 0.47	mg C1/g E dw
		C2				3.95 ± 0.24	mg C2/g E dw
		CT				11.76 ± 0.82	mg CT/g E dw

## Supplementary Materials

The supplementary materials are available online.
