# Supplementary material for: Ultrasound as a Rapid and Low-Cost Extraction Procedure to Obtain Anthocyanin-Based Colorants from Prunus spinosa L. Fruit Epicarp: Comparative Study with Conventional Heat-Based Extraction
Source: Molecules. 2019 Feb 5;24(3):573. doi: 10.3390/molecules24030573 (PMC6384548; doi:10.3390/molecules24030573)
Supplement: Supplementary file 1 [file molecules-24-00573-s001.pdf]

Supplementary Material

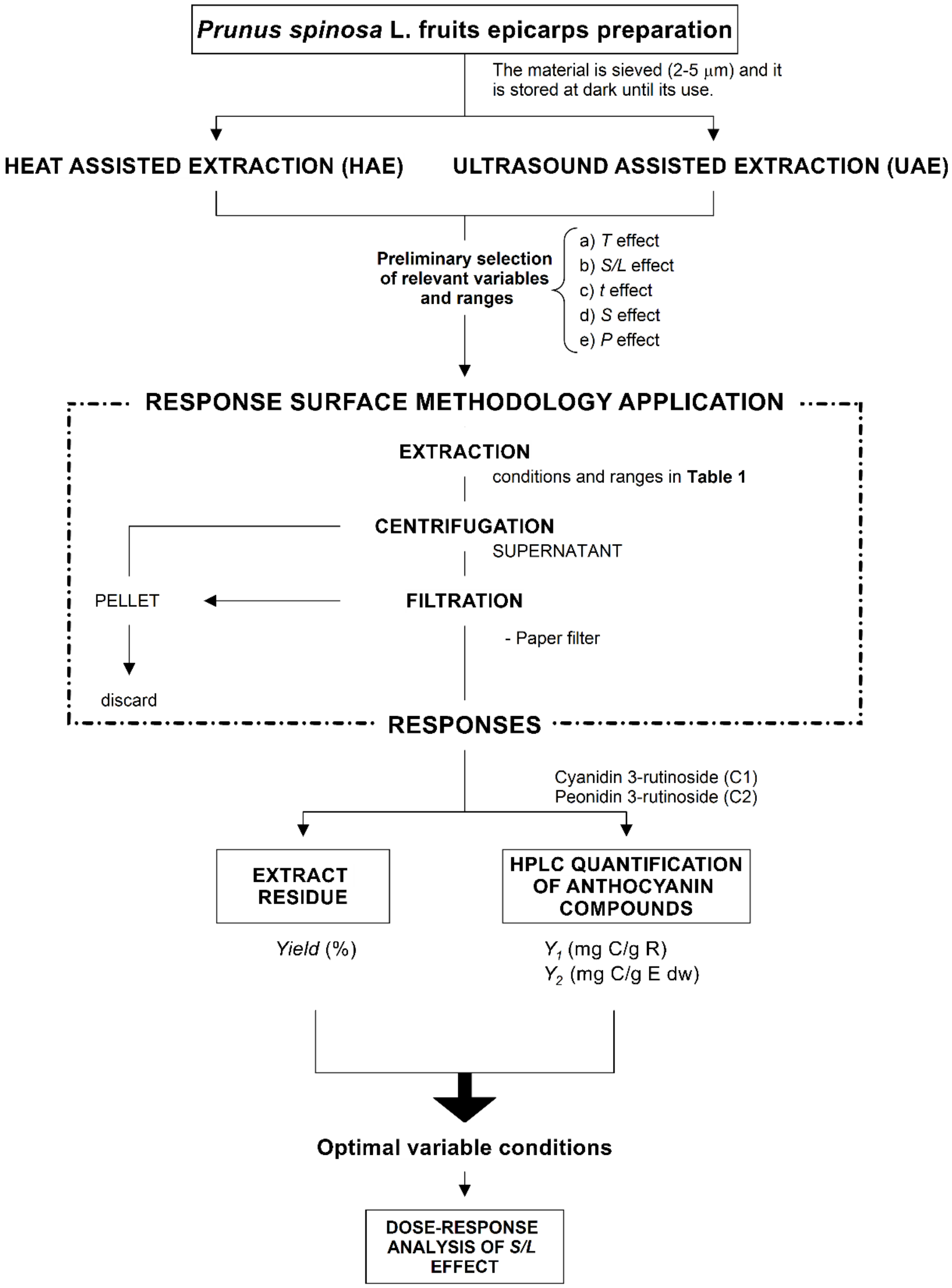

Figure A1: Diagram of the different steps carried out for optimizing the conditions that maximize the extraction responses (Y_1, mg C/g R and Y_2, mg C/g E dw) of the anthocyanin compounds and the total extracted residue (*Yield*, %).

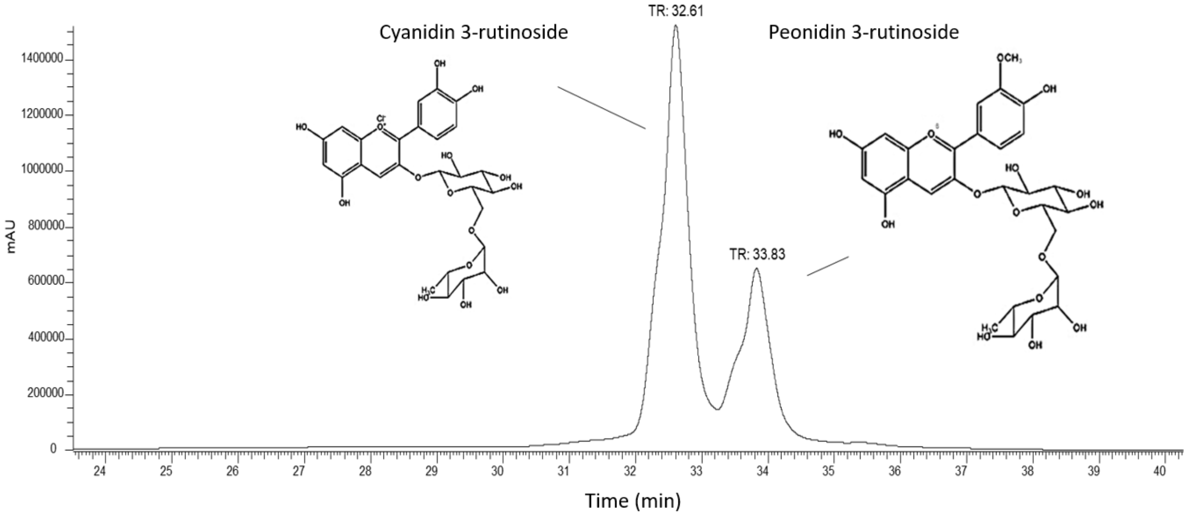

Figure A2: Chromatographic example of HPLC-DAD-ESI/MS results for the quantification of anthocyanin compounds of cyanidin 3-rutinoside ($[M+H]^+$ at *m/z* 595) and peonidin 3-rutinoside ($[M+H]^+$ at *m/z* 609) in *P. spinosa* fruits.

Table A1: Mathematical models of the extraction process derived from the second-order polynomial model with interaction described in Eq. **Error! Reference source not found.**.

**For the Y_1 response format (mg C/g R):**			
**HAE**	Cyanidin 3-rutinoside (C1):	$HAE^{C_1}_{Y_1} = 9.35 + 0.33t + 0.56S - 0.26t^2 - 1.60S^2 + 0.26tS + 0.31TS$	Eq. (2)
	Peonidin 3-rutinoside (C2):	$HAE^{C_2}_{Y_1} = 4.15 + 0.33S - 0.09t^2 - 0.69S^2 + 0.04tS + 0.14TS$	Eq. (3)
	CT (C1+C2):	$HAE^{C_T}_{Y_1} = 13.48 + 0.28t + 0.88S - 0.35t^2 - 2.26S^2 + 0.31tS + 0.45TS$	Eq. (4)
**UAE**	Cyanidin 3-rutinoside (C1):	$UAE^{C_1}_{Y_1} = 10.42 + 0.23P - 0.36S^2 - 0.34tP + 0.17PS$	Eq. (5)
	Peonidin 3- rutinoside (C2):	$UAE^{C_2}_{Y_1} = 4.10 + 0.18P - 0.16S + 0.26t^2 + 0.35P^2 - 0.84S^2 - 0.11tP + 0.22PS$	Eq. (6)
	CT (C1+C2):	$UAE^{C_T}_{Y_1} = 14.46 + 0.37P + 0.66P^2 - 1.22S^2 - 0.46tP + 0.41PS$	Eq. (7)
**For the Y_2 response format (mg C/g E dw):**			
**HAE**	Cyanidin 3-rutinoside (C1):	$HAE^{C_1}_{Y_2} = 5.07 + 0.25t + 0.15T + 0.17S - 0.10t^2 - 0.94S^2 + 0.18tS + 0.21TS$	Eq. (8)
	Peonidin 3-rutinoside (C2):	$HAE^{C_2}_{Y_2} = 2.26 + 0.05T + 0.11S - 0.05t^2 - 0.40S^2 + 0.04tS + 0.09TS$	Eq. (9)
	CT (C1+C2):	$HAE^{C_T}_{Y_2} = 7.29 + 0.26t + 0.19T + 0.27S - 0.16t^2 - 1.33S^2 + 0.22tS + 0.29TS$	Eq. (10)
**UAE**	Cyanidin 3-rutinoside (C1):	$UAE^{C_1}_{Y_2} = 6.98 - 0.13t + 0.25P - 0.55S - 0.13t^2 - 1.02S^2 - 0.18tP - 0.08PS$	Eq. (11)
	Peonidin 3-rutinoside (C2):	$UAE^{C_2}_{Y_2} = 2.83 - 0.10t + 0.14P - 0.26S + 0.19P^2 - 0.68S^2 - 0.07tP$	Eq. (12)
	CT (C1+C2):	$UAE^{C_T}_{Y_2} = 9.75 - 0.22t + 0.36P - 0.82S + 0.27P^2 - 1.84S^2 - 0.23tP$	Eq. (13)
**For the *Yield* response format (%):**			
**HAE**	*Yield:*	$HAE_{Yield} = 54.86 + 1.54t + 3.12T - 3.07S - 1.24S^2 + 0.78tT + 0.54tS + 0.69TS$	Eq. (14)
**UAE**	*Yield:*	$UAE_{Yield} = 68.11 - 1.70t + 2.12P - 6.46S - 2.29t^2 - 7.33S^2 - 1.29PS$	Eq. (15)
